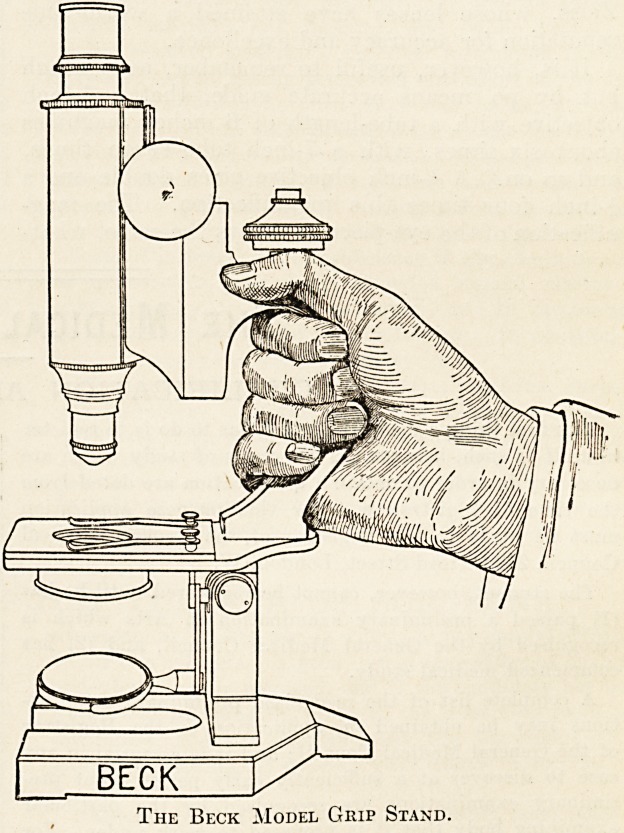# How to Choose and Use a Microscope

**Published:** 1911-09-02

**Authors:** 


### HOW TO CHOOSE AND USE A MICROSCOPE.

At the outset it is necessary to warn the prospective buyer of a microscope against being too modest in his demands regarding the capability and adaptability of the instrument he is about to choose. The remarks in this article are specially intended for those who require a microscope for their medical studies and for use afterwards in practice. Even if the latter provision appear to have only a remote chance of being fulfilled, it must be allowed that for medical studies only no instrument which cannot be made equal to the demands of ordinary bacteriological work should be considered. The medical student is ill-advised who purchases at the beginning of his career a microscope only barely sufficient for examining sections; later he finds that he needs a better instrument and has to lay out a large sum, towards which the sale of his first microscope provides a disappointingly small amount. It may seen unnecessary to mention so obvious a truth, but such improvident mistakes are sometimes made. As a rule the teachers in the medical schools take the trouble to advise their classmen early in the session and will examine instruments before their purchase is concluded. Over-elaboration in the matter of accessories is not so commonly met with except perhaps in "presentation" microscopes, but this may be a hindrance while working at preliminary studies and, moreover, does not conduce to speedy work, while the finer accessories are likely to suffer from rough usage and to become defective by the time they are really required.

It may, however, be broadly stated that since a microscope is in the great majority of cases purchased only once in a lifetime it is generally advisable not to economise on the stand, but rather to cut down the initial optical equipment and to supplement it at a future date. There is an almost embarrassing choice presented in the catalogues of the reputable makers, and as this choice of a microscope must necessarily be largely governed by the means at the disposal of the student, it is desirable to have a clear understanding that certain qualities and accessories are essential for useful work. Out of the multiplicity of possible combinations we may here indicate, as a sort of ground plan, which may be added to, the desirable features of a good instrument before entering on a discussion of the several details.

The stand should be strong and very stable, and the type should be the single tube monocular form, with rack and pinion coarse adjustment and draw tube. The fine adjustment is of the greatest importance and must be of first-class workmanship, durable and very sensitive. A dust-proof triple nose-piece is to be recommended; although only two objectives may be purchased at the outset, a third is sure to be required later. An Abbé condenser with adjustment for raising and lowering, an iris diaphragm, and a double mirror will also be included. Two eye-pieces will be sufficient, and for objectives, a $\frac{2}{3}$ inch or a $\frac{3}{4}$ inch and a $\frac{1}{6}$ inch will do at the commencement of medical studies, but a $\frac{1}{12}$ inch oil-immersion will certainly be a necessity at a later stage. Now that it is possible to obtain much more satisfactory detachable mechanical stages than was formerly the case, it becomes a moot point whether this accessory should be included in the initial outlay; the advantages of a mechanical stage will be evident as soon as hæmatology and bacteriology are being studied, and although a detachable mechanism leaves the stage free for Petri dishes, etc., there are several models with incorporated mechanical stages in which no disadvantage exists in this respect. It is therefore worth while to allow for this additional expense or to obtain a model to which a good mechanical stage can afterwards be accurately fitted.

Some of these items may now be considered in more detail. The form of stand recognised as being the steadiest is that with the tripod foot, and this is also lighter than the horseshoe base. The steadiness of the instrument is obtained by the spread of the foot and by balance. The horseshoe foot may be more compact, and where portability is a consideration it has advantages. The body may be hinged, so as to be inclinable if desired, but if so it is well to see that there is present a clamp which will fix the instrument in the inclined position. A very useful modification of the usual shape of stand is found in the "London" models of Messrs. Beck of Cornhill. In these instruments the limb of the microscope has been shaped so as to form a handle for lifting. This handle is fixed directly on to the stage, and thus none of the adjustments are in danger of shifting when the instrument is picked up by the handle. Some of the models made by Carl Zeiss, and also some by Mr. C. Baker—notably the D.P.H. 1a model—have a convenient handle incorporated in the stand, by which the whole may be safely lifted.

Too much importance can hardly be attached to the workmanship of the fine focussing mechanism, and much care has been bestowed by instrument-makers in perfecting this. The lever principle is employed in many types, and the fineness of the adjustment depends on the delicacy of a micrometer screw which, impinging on a lever, actuates through springs the motion of the body of the microscope. In a new series of models by Leitz, of Oxford Street, the fine focussing adjustment is in the form of a continuous spiral cam and worm screw. With a good type of fine adjustment it should be almost impossible to break a cover-glass or damage the front lens of the objective, because when they are brought in contact only the weight of the body with the pressure of a light spring is resting on the cover-glass, and this is not sufficient to do damage. A double-speed fine adjustment is fitted to the "Imperial" model made by Beck, and this enables one by a simple arrangement to use either a comparatively coarse "fine" adjustment with moderate powers or a very fine adjustment with the highest powers. A slow motion fine enough for focussing the highest powers is troublesome when working with moderate power lenses. Instead of being placed at the top of the pillar the fine adjustment screw is sometimes placed low down on the body, or even at the bottom of the horizontal limb, anterior to the main pillar, as in some of Baker's models, a position that will be found very comfortable to the worker, as well as sheltering the screw from accidental damage.

The substage condenser is a system of lenses placed immediately below the object, to throw a wide-angled cone of light upon the object under examination. The so-called Abbé condenser is not fully corrected either for chromatic or spherical aberration, and although sufficient for ordinary work, even with high powers, it is not desirable for the formation of critical images; but, on the other hand, the Abbé condenser is perhaps the most efficient form for dark-ground illumination with suitable stops. An achromatic combination of lenses for the substage condenser will give better results with high powers, but is, of course, more expensive. Concerning the substage mechanism, it is only necessary to add that it should have a rack and pinion coarse adjustment and be provided with centring screws; a fine adjustment here adds an expense which cannot be regarded as essential for ordinary work. When using a high power, accurate centring of the condenser is of great importance, and the condenser itself will have to be racked up close to the glass slide under examination. It will be found convenient to have the substage so arranged as to be capable of being swung aside at will, as when using low powers. An "iris" diaphragm is not only neater than a series of stops, but also more effectively and easily controlled. Under the diaphragm will often be provided a ring, which is grooved for the purpose of holding a ground-glass or coloured screen. The mirror will be flat on one side, for use with the condenser, and concave on the other; it should be freely and smoothly movable in all directions, and detachable.

The patterns and modifications of the stage itself are very numerous. We urge the student to favour simplicity and an unencumbered stage. Two spring clips for holding the slide should be readily removable, and the milled screws belonging to the mechanical stage should be well away from the edge of the stage, so as to give freedom for the hand and to allow the placing of culture plates, etc., under the objective. If an attachable mechanism is to be purchased separately, it must be carefully seen that the stage is adapted for that purpose; when the addition is needed it is better to send the microscope back to the makers to have this accessory perfectly adapted, for it is absolutely essential that the working of the stage should be quite smooth and sensitive to small movements. We cannot here go into the details of mechanical stages; different patterns are associated with the several models, the choice of the latter being the determining factor. It may be considered an advantage to have an extra milled head on the left-hand side of the stage in order that motion in one direction at least can be controlled by the left hand. As for attachable mechanical stages, in which great improvements have been made of recent years, we may say that it is necessary to get a well-finished article, for the rack and pinion work will have to stand a good deal of wear. A pattern such as the "Mayall," made by James Swift & Son, with the milled screws projecting on fairly long arms, is a satisfactory one; but there are many patterns to be had which give a sufficiently wide range of movement and fit neatly to an ordinary stage. Those mechanisms depending on the friction of small wheels against the edge of the slide are not recommended, as in practice the wheels soon begin to slip, and then motion in one direction becomes defective and irregular. We have already indicated that the objectives required

The Beck Model Grip Stand.

will be a $\frac{2}{3}$ inch or a $\frac{3}{4}$ inch, a $\frac{1}{6}$ inch and a $\frac{1}{1}$ inch oil-immersion. The latter will, however, not be needed in the preliminary studies. These lenses are subjected to rigorous tests before leaving the makers, but it is a good plan to try them before purchase. Some trial of their qualities of flatness of field and of definition may be made on a suitable blood-film preparation, and in this the novice should obtain the services of an expert pathologist. When fitted to a revolving nose-piece, the objectives ought to come approximately into focus; but this detail is not of very great importance. In choosing the $\frac{1}{6}$ inch power it is very desirable to see if it will focus through the thinner of the two cover-glasses of the Thoma-Zeiss hæmacytometer; if this is done, much annoyance and disappointment will be saved in the future. A $\frac{1}{6}$ inch with sufficient working distance to focus through the thicker cover-glass can be obtained, if specially desired, and generally without extra cost.

The subject of eye-pieces and their relation to the several objectives is a highly technical one and beyond the scope of this article. Suffice it to say that there is no fixed nomenclature for eye-pieces, and their numbering varies with different makers. The firm supplying the microscope will fit those eye-pieces corresponding to the type of objective and the magnification desired. For ordinary purposes one eye-piece can be made to work satisfactorily with all three objectives, and more than two will scarcely be required. For information on apochromatic objectives and Huyghensian oculars the student cannot do better than consult the instructive catalogue issued by Carl Zeiss, whose lenses have attained a world-wide reputation for accuracy and excellence.

It is, however, useful to remember, as a rough but by no means accurate guide, that a 1-inch objective with a tube-length of 6 inches magnifies about six times, with a 7-inch tube seven times, and so on. A $\frac{1}{2}$-inch objective gives double and a $\frac{1}{4}$-inch four times this magnification. The magnification of the eye-piece is always the same, whatever the length of the tube, as this part of the optical arrangement only magnifies the virtual image formed by the object-glass. This magnification is dependent on the focal power of the eye-piece, and the figure varies with the numbering employed by the different makers. A useful eye-piece is one magnifying about seven or eight times. By multiplying the power of the objective by that of the eye-piece the total magnification is obtained.

In conclusion we may remark that the choice of a microscope is not an easy matter, and it is practically impossible to be sure one has chosen the best instrument for one's individual requirements unless one can see a considerable number of patterns side by side. Many of the makers are willing to afford opportunities of such comparison of their own models with those of other firms. It will be found that every assistance will be given in this direction to students by the best firms, and thus a selection can be comfortably made from the numerous models specially adapted for their requirements sold by such firms as Messrs. Beck, of 68 Cornhill; E. Leitz, of Oxford House, 9 Oxford Street, W.; James Swift & Son, 81 Tottenham Court Road; C. Baker, of 244 High Holborn; and Carl Zeiss, of 13 Great Castle Street, Oxford Circus.

## Figures and Tables

**Figure f1:**